# Surface Roughness-Controlled Sinter-Bonding of Binder-Free Ag Preforms Consolidated Directly from Ag Nanoparticles

**DOI:** 10.3390/mi17101167

**Published:** 2026-10-07

**Authors:** Hoi-Woon Lee, Jong-Hyun Lee

**Affiliations:** 1 Department of Materials Science and Engineering, Seoul National University of Science and Technology, Seoul 01811, Republic of Korea; 2 Research Institute for Future Convergence Materials, Seoul National University of Science and Technology, Seoul 01811, Republic of Korea

**Keywords:** Ag nanoparticles, sinter-bonding, pressure-assisted sintering, die-attach, interfacial connection ratio, power module packaging

## Abstract

Ag sinter-bonding is the mainstream die-attach technology for SiC power modules; however, organic residues from pastes and binder-containing sheets can degrade the thermal conductivity and reliability of the bond line. In this study, a binder-free porous Ag preform was fabricated by direct hot pressing of in-house-synthesized, quasi-spherical Ag nanoparticles (70.2 nm) at 200 °C for 1 min under a pressure of 10 MPa to serve as the interlayer. To evaluate its performance, the preform was sandwiched between the Ag-electroplated Cu chips and substrates, and their sinter-bonding behavior was evaluated at 250 °C under a chip-compatible pressure of 10 MPa in air. The surface roughness of the preform, controlled by lapping, was found to be a key factor governing the bonding performance. A precision-lapped preform (Ra = 35.8 nm), smoother than the electroplated Ag layers, allowed the asperities of the counterparts to be plastically flattened under pressure, maximizing the real interfacial contact area. Consequently, the shear strength reached 28.1 MPa after only 1 min and increased to 57.6 MPa after 3 min, with the interfacial connection ratio increasing from 62.9% to 82.5% and the bond-line porosity decreasing to 10.4%. TEM and HAADF-STEM analyses verified that oxide- and void-free metallurgical bonding was formed by the interdiffusion of Ag atoms across the preform/Ag layer interface. Fractographic analyses confirmed cohesive failure with highly stretched metallic ligaments, corroborating the formation of sound bond lines. The developed nanoparticle-derived preform is a promising die-attach solution that combines high-temperature reliability with rapid low-pressure processability.

## 1. Introduction

The rapid electrification of transportation, represented by electric vehicles, the continuous emergence of applications, such as robotics, and the rapid growth of data centers have increased the demand for electric power requiring conversion, control, and storage. Consequently, the development of high-efficiency power-conversion modules has received considerable attention worldwide [1,2,3]. To meet these demands, wide-bandgap semiconductors, particularly SiC, have been rapidly adopted because they offer higher breakdown voltages, lower switching losses, and higher permissible operating temperatures than conventional Si devices, enabling miniaturization and weight reduction in power modules [4,5,6]. Furthermore, owing to the excellent thermal conductivity of SiC, its integration with packaging materials and architectures that provide superior heat dissipation can simplify or even eliminate the need for cooling modules. This provides additional competitiveness for portable and weight-sensitive power applications [7,8]. However, the SiC device junction temperatures can exceed 200 °C, imposing severe requirements on the packaging materials. In particular, the chip-attach layer, which provides thermal, electrical, and mechanical connections between the chip and substrate, is directly exposed to the highest temperature in the package and must, therefore, simultaneously ensure high-temperature reliability and excellent thermal conductivity [6,9]. Conventional Sn-based solders cannot satisfy these requirements because their melting points are close to the operating temperatures of SiC devices, resulting in severe strength degradation and accelerated thermal fatigue [10,11,12]. Consequently, sinter-bonding using Ag particles, which forms a bond line whose melting point (961 °C) is far above the bonding temperature and which possesses the highest thermal conductivity among metals, has become the mainstream die-attach technology for SiC power modules [8,9,13,14].

Industrially, Ag sinter-bonding has mostly been implemented using pastes in which Ag particles (mainly nanoparticles) are dispersed in organic vehicles [15,16]. Such pastes provide good printability and initial conformity to faying surfaces; however, the organic components must thermally decompose and evaporate during bonding, and their incomplete removal leaves organic residues and pores in the bond line, degrading their thermal and mechanical properties [8,17,18]. In addition, the removal of organics requires additional pre-drying steps and prolonged heating profiles; when the process is accelerated, the abrupt outgassing of organics generates large voids within the bond line [18,19,20]. The rheology and storage stability of the paste must also be strictly controlled because solvent evaporation during storage and printing alters the paste behavior and, consequently, the reproducibility of the bond line thickness and quality [21,22].

As an alternative that can mitigate these paste-related issues, Ag sheets (or films), i.e., free-standing preform-type materials, have been increasingly employed in recent years [14,23]. Because no printing or pre-drying step is required, and a uniform and well-defined bond-line thickness can be readily achieved by simply placing a sheet stamp matched to the chip size between the chip and substrate, they are particularly attractive for mass production [14,23]. Nevertheless, currently available Ag sheets are not free from these limitations. Their fabrication processes are demanding and complicated, and multiple organic components such as binders must be incorporated to realize the sheet form, which can remain within the bond line even after sinter-bonding, potentially reducing the thermal conductivity and reliability of the bond line [24]. Accordingly, if a preform-type die-attach material could be fabricated through a simple consolidation route without the deliberate addition of binder-type organics while retaining high sintering activity, it would inherit the handling and mass-production advantages of the sheet approach without its drawbacks.

The excellent sinter-bondability of Ag nanograins has only recently been quantitatively verified. Chen et al. reported remarkable shear strengths of 79.8 and 87.4 MPa for an organic-solvent-free Ag preform after sinter-bonding at 250 °C under 20 MPa for 1 and 3 min, respectively [23]. Instead of Ag nanoparticles, the preform was fabricated by hot-pressing ball-milled Ag flake particles at 300 °C under 20 MPa. The Ag flakes, endowed with a high dislocation density by ball milling, underwent accelerated interparticle neck growth and thus effectively formed the preform. Subsequent surface polishing transformed the preform surface into ultrafine grains and generated surface-driven stress. Consequently, the formation of compressive residual stress at the preform surface greatly accelerated the diffusion of Ag and drove abnormal grain growth. This induced grain integration with the contacting Ag layer beneath the SiC chip, thereby enabling sinter-bonding within a short time [23]. However, this preform fabrication route relies on ball milling to produce Ag flake particles with high dislocation densities, which makes it difficult to ensure uniform flake quality. Even slight pressure welding between particles during milling is expected to affect the subsequent preform characteristics. In addition, the applied pressure of 20 MPa employed in sinter-bonding is a rather excessive process condition that may damage the SiC chips.

Therefore, in this study, rather than inducing the transition from high-dislocation-density Ag flake particles to ultrafine grains, the as-prepared Ag nanoparticles were directly consolidated into a preform, and their sinter-bonding performance was evaluated. Because nanoparticles inherently possess high surface energies and short diffusion distances, this fabrication route provides the opportunity for activated sintering behavior to proceed uniformly throughout the entire preform, together with the fabrication of a more stabilized preform, thereby contributing to the realization of more consistent bonding characteristics. Furthermore, sinter-bonding was performed under an applied pressure of 10 MPa to analyze whether the bonding results were compatible with the actual bonding of SiC chips.

## 2. Materials and Methods

### 2.1. Synthesis of Ag Nanoparticles

The Ag nanoparticles used for preform fabrication were synthesized in-house using the following procedure. A silver precursor solution was prepared by completely dissolving 6.56 g of silver nitrate (AgNO_3_, 99.99%, Hojeonable Inc., Daejeon, Republic of Korea) in 107.5 mL of deionized (DI) water, and a 17.5 mL potassium hydroxide solution was separately prepared by dissolving a small amount of potassium hydroxide (KOH, 99.99%, Duksan Pure Chemical Co., Ltd., Ansan-si, Republic of Korea) in DI water. Polyvinylpyrrolidone (PVP; molecular weight: 30,000 g/mol; Duksan Pure Chemical Co., Ltd., Ansan-si, Republic of Korea) was added to the Ag precursor solution and thoroughly mixed by stirring, after which the prepared KOH solution was poured into the precursor solution and stirred for 10 min. Finally, 100 mL of hydrogen peroxide (H_2_O_2_, 30%, Daejung Chemicals & Metals, Siheung-si, Republic of Korea) was added to the mixed solution to reduce the silver ions. After completion of the reaction, the resulting slurry was subjected to three consecutive cycles of centrifugation, decanting, and acetone rinsing, followed by a final ethanol rinse. The nanoparticles were collected by centrifugation. The obtained nanoparticles were then dried in a vacuum chamber at room temperature for 6 h.

The morphology of the synthesized Ag nanoparticles was observed using scanning electron microscopy (SEM, SU8010, Hitachi High-Technologies, Tokyo, Japan), and the average particle size was measured using an image analysis program (ImageJ 1.54, National Institutes of Health, Bethesda, MD, USA). The phases of the nanoparticles were analyzed using X-ray diffraction (XRD, D8 Advance, Bruker, Karlsruhe, Germany), their thermal behavior was analyzed using thermogravimetric–differential thermal analysis (TG-DTA, DTG-60H, Shimadzu, Kyoto, Japan), and their surface chemical states were determined using X-ray photoelectron spectroscopy (XPS, K-Alpha+, Thermo Fisher Scientific, Waltham, MA, USA).

### 2.2. Preparation of Ag Preform

**Figure 1:**
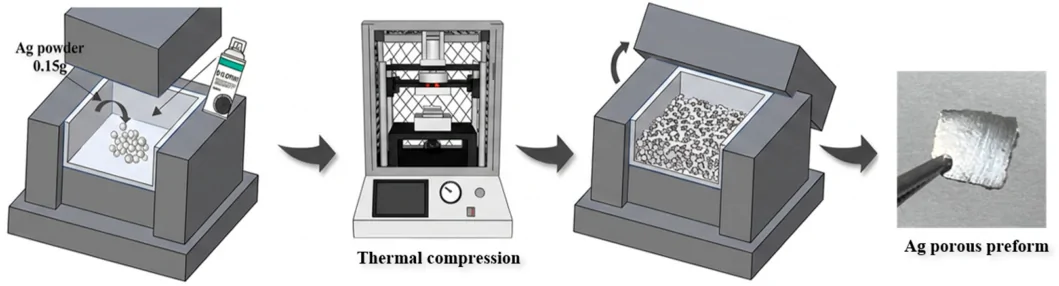
Schematic illustration of the fabrication of the Ag preform.

The synthesized Ag nanoparticles were placed in a custom-made mold and hot-pressed at 200 °C for 1 min under a pressure of 10 MPa, as illustrated in Figure 1. This process was intended to form a porous microstructure that retains the shape of the preform while avoiding excessive densification; therefore, sinter-bonding through the rearrangement of the constituent nanoparticles remains possible during subsequent thermal compression. To prevent the nanoparticles from adhering to the mold during hot pressing, a boron nitride (BN) spray (Nabakem, Nambang CNA Co., Ltd., Pyeongtaek-si, Republic of Korea) was applied to the inner walls of the mold before loading the nanoparticles.

Both faces of the 325-μm-thick hot-pressed Ag preform were mechanically lapped either manually on 2000-grit sandpaper or using a precision lapping fixture (PELCO 15000 Micrometer Lapping Fixture, Ted Pella, Inc., Redding, CA, USA) to remove the spray residue and to control the surface roughness. The average thickness of the lapped Ag preform was 275 μm. The surface states of the lapped Ag preforms were observed using SEM, and the quantitative surface roughness (R_a_) was measured using a 3D confocal laser scanning microscope (LSM 800 MAT, Carl Zeiss Microscopy GmbH, Jena, Germany).

### 2.3. Characterization

To evaluate the sinter-bonding characteristics of the fabricated Ag preform, thermo-compression die-attach experiments were conducted in air using a dummy chip and substrate made of Cu plates electroplated with a 1-μm-thick Ag layer. The Ag preform was cut into 4 mm × 4 mm pieces and placed on the Ag-electroplated Cu substrate (10 mm × 10 mm × 1 mm). A 3 mm × 3 mm × 1 mm Ag-electroplated Cu chip was then placed on top to form a sandwich-structured sample. The prepared sample was heated to 250 **°**C at a rate of approximately 30 **°**C/s under an applied pressure of 10 MPa and then sintered for a given time. The bonding time of 1–3 min discussed in this study includes the heating time. Accordingly, the dwell times at 250 °C under the 1-min and 3-min bonding conditions were 52.5 and 172.5 s, respectively. The cross-sectional microstructures of the formed bond lines were observed by SEM after polishing. SEM micrographs were analyzed using ImageJ software (National Institutes of Health, Bethesda, MD, USA) to quantitatively determine the porosity within the formed bond line. The sinter-bonded interface between the Ag preform and Ag-plated Cu chip was examined using a transmission electron microscope (TEM) (JEM-2010, JEOL Ltd., Tokyo, Japan). In addition, high-angle annular dark-field scanning transmission electron microscopy (HAADF-STEM, NEO-ARM, and JEOL) was used to analyze the interfacial microstructure in greater detail.

The strength of the formed bond lines was evaluated as a function of bonding time by shear testing using a micro-bond tester (Dage-4000, Nordson DAGE, Aylesbury, UK). The shear strength was defined as the maximum stress recorded as the shearing tip traveled at a speed of 200 μm/s at a height of 200 μm above the substrate surface. Measurements were performed on 10 samples for each condition, and the mean value was taken as the shear strength. The fracture surfaces after the shear tests were examined in detail using SEM.

## 3. Results and Discussion

### 3.1. Characteristics of the Ag Nanoparticles

**Figure 2:**
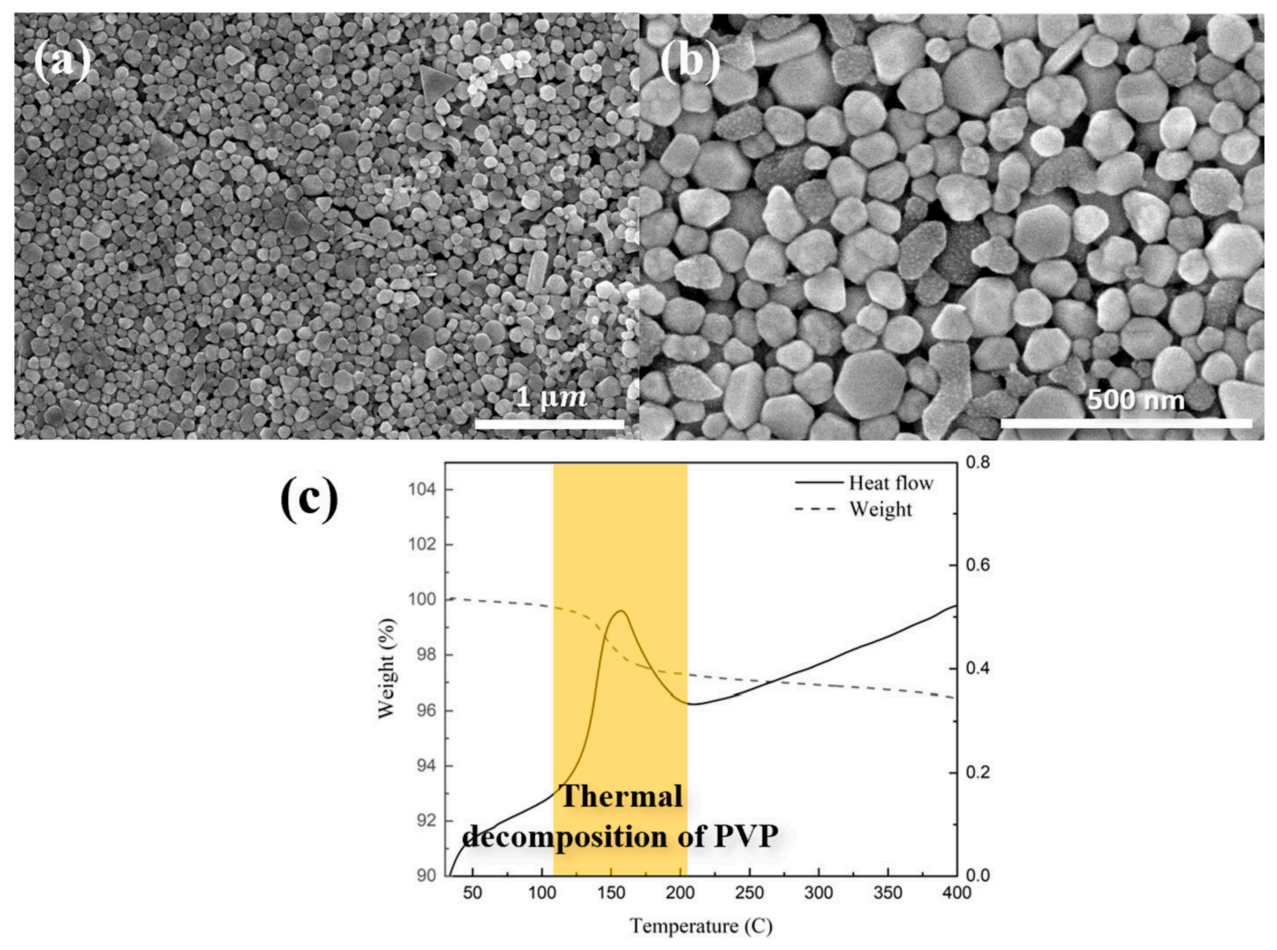
(**a**) Low- and (**b**) high-magnification SEM images and (**c**) TG-DTA results of the synthesized Ag nanoparticles.

Figure 2 shows SEM images of the nanoparticles synthesized in this study using PVP as a capping agent. The synthesized particles exhibited a quasi-spherical morphology with an overall uniform size and existed in a relatively well-dispersed state without pronounced interparticle aggregation or abnormal growth. Although nanoparticles smaller than 100 nm generally tend to aggregate easily because of their high surface energy, the present results are attributed to the effective adsorption of PVP on the particle surfaces. This PVP adsorption effectively prevents interparticle aggregation and helps maintain the well-dispersed state of the nanoparticles [25,26]. Particle size analysis revealed an average size of 70.2 (±6.31) nm. Nanoparticles of such a uniform size exhibit excellent dispersibility during preform preparation, and during subsequent sintering, their high surface energy and short diffusion distances are expected to facilitate effective interparticle neck formation, rapid densification under pressure, and excellent bonding characteristics. Figure 2c shows the TG-DTA results for the synthesized nanoparticles. An exothermic peak was observed at approximately 150 °C together with weight loss during heating, which was attributed to the thermal decomposition of the PVP coating the nanoparticle surfaces and concurrent exothermic sintering between the contacting nanoparticles [18]. Thereafter, the thermal decomposition of PVP continued gradually, resulting in a total weight loss of approximately 4.7% upon heating to 400 °C. Because no weight gain due to the oxidation of Ag occurs in air [27], the observed weight loss of 4.7% is interpreted as the total content of capping PVP present on the nanoparticle surfaces.

**Figure 3:**
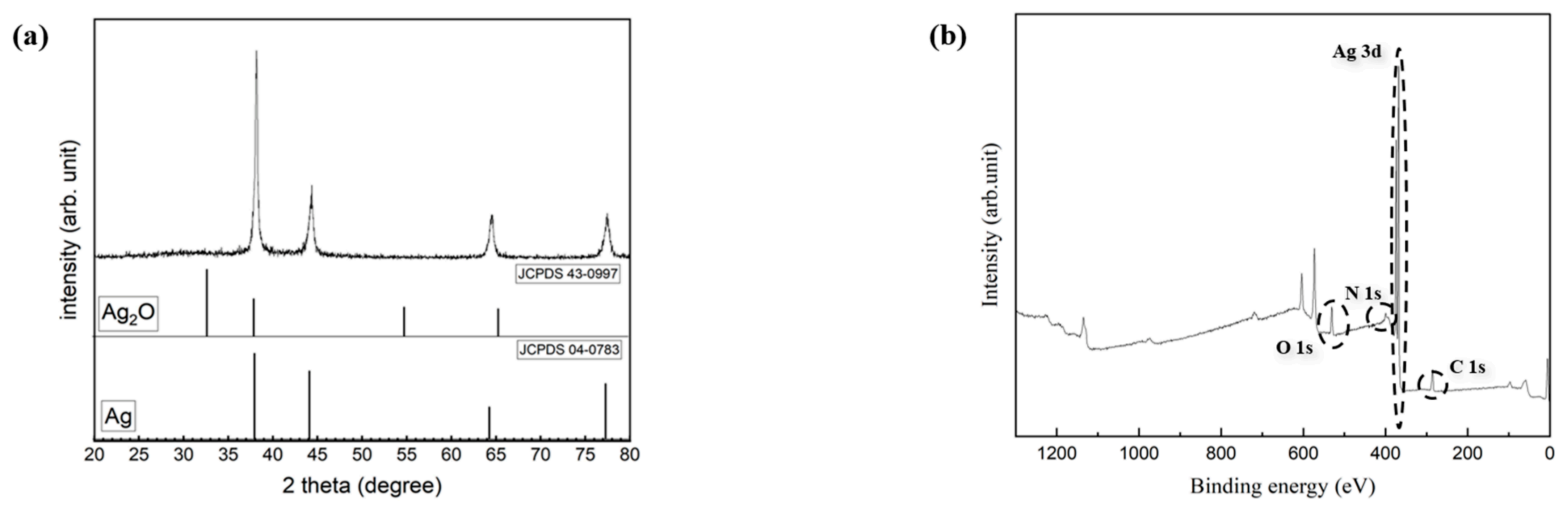
(**a**) XRD pattern and (**b**) XPS survey spectrum of the synthesized Ag nanoparticles.

Figure 3 shows the XRD patterns and XPS results for the synthesized nanoparticles. In the XRD pattern in Figure 3a, distinct diffraction peaks corresponding to the (111), (200), (220), and (311) planes of the Ag phase (JCPDS Card No. 04-0783) appeared at 2θ = 38.1°, 44.3°, 64.4°, and 77.5°, respectively [28,29]. In contrast, no additional diffraction peaks originating from silver oxide phases such as Ag_2_O were observed, confirming that pure crystalline Ag nanoparticles were successfully synthesized without the formation of oxide phases. In the XPS survey spectrum shown in Figure 3b, the C 1s, N 1s, and O 1s peaks originating from PVP were identified together with the Ag 3d peaks [30]. The C 1s and N 1s peaks correspond to the carbon backbone of PVP and the nitrogen atoms in the pyrrolidone ring, respectively [27,31], whereas the O 1s peak originates from the oxygen in the carbonyl group of PVP [27,31]. The simultaneous detection of these PVP-derived elements indicated that PVP remained on the Ag nanoparticle surfaces even after synthesis. In particular, the carbonyl oxygen of PVP is considered to interact with and adsorb onto the Ag surfaces to form a capping layer [26,27]. Owing to this stable PVP capping, aggregation-free nanoparticles can be obtained, as shown in Figure 2a,b.

### 3.2. Structure and Surface Roughness of the Ag Preform

**Figure 4:**
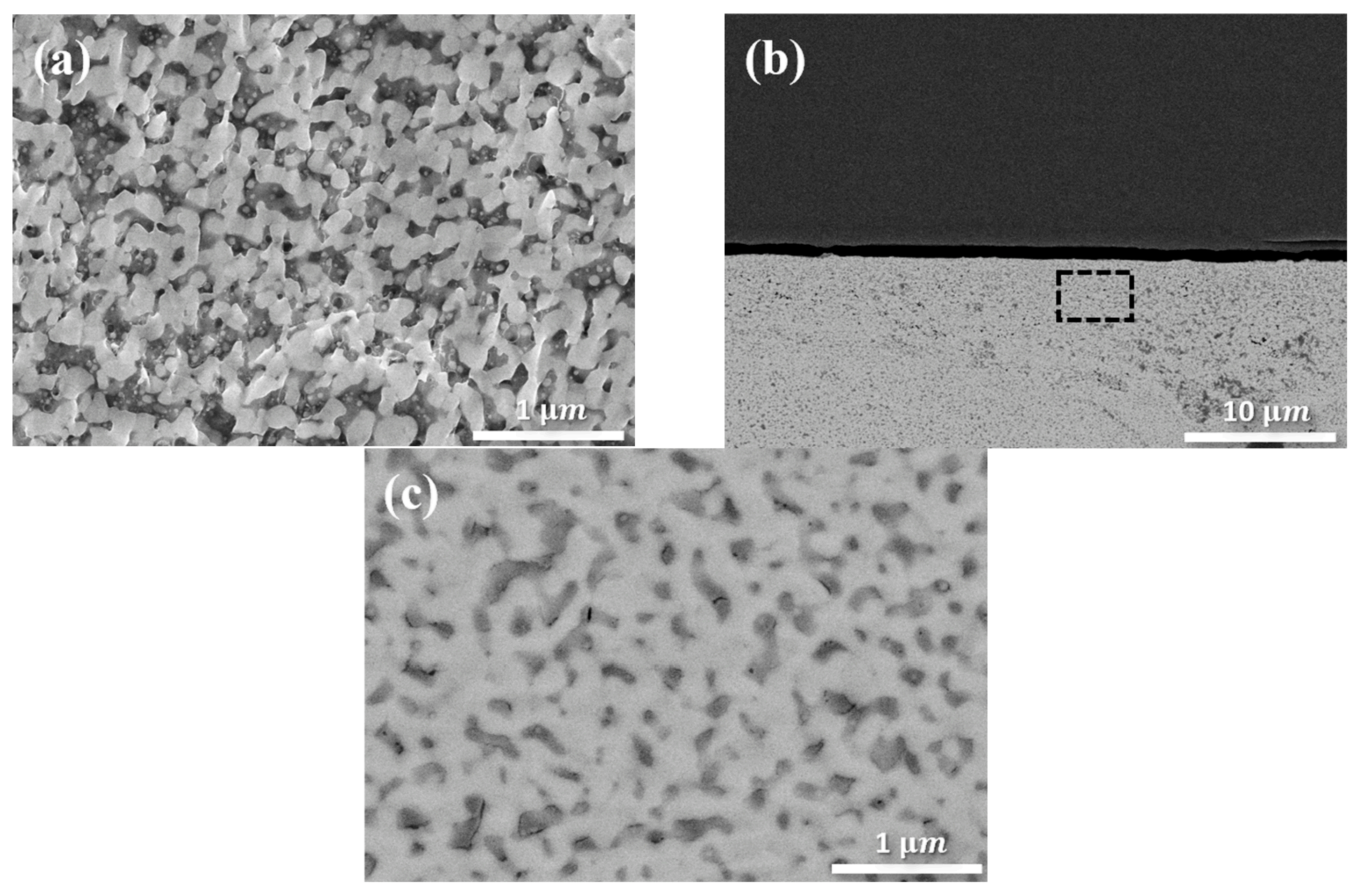
(**a**) Surface SEM image of the preform fabricated by hot pressing at 200 °C for 1 min, (**b**) cross-sectional BSE image of the preform, and (**c**) enlarged view of the near-surface region in (**b**).

Figure 4 shows SEM images of the surface and cross-sectional microstructures of the Ag preform fabricated by hot pressing at 200 °C for 1 min. Figure 4a shows the surface morphology of the fabricated preform, confirming that the Ag nanoparticles were sintered to the extent that they formed necks with one another, resulting in a porous network structure. Figure 4b shows a cross-sectional BSE image of the preform, while Figure 4c shows an enlarged view of the dotted rectangular region in (b), indicating that a relatively uniformly distributed porous structure was also formed inside the preform. This porous structure is considered to have formed through limited sintering centered on the contact points between the Ag particles as the capping PVP decomposed and was removed during preform fabrication. Such a porous structure enables local plastic deformation when an external pressure is applied during subsequent pressure-assisted bonding, effectively accommodating the fine surface asperities of the counterpart faying surfaces and thereby increasing the real contact area. In particular, the mixed structure of ~70 nm Ag particles and the voids formed among them are expected to conform even to asperities of several tens of nanometers on the faying surfaces, minimizing unbonded interfacial regions that may arise in the initial contact stage and contributing to a more uniform interfacial contact overall. Consequently, a high interfacial connection ratio between the preform and faying surfaces can be secured, which is expected to result in stable interface formation and, ultimately, excellent mechanical properties of the joint. Because such interfacial conformability is anticipated to be a particularly important factor in inducing sinter-bonding within a short time—the objective of this study—the interfacial contact characteristics and the resulting sinter-bonding behavior were evaluated as a function of the initial surface asperities of the preform.

**Figure 5:**
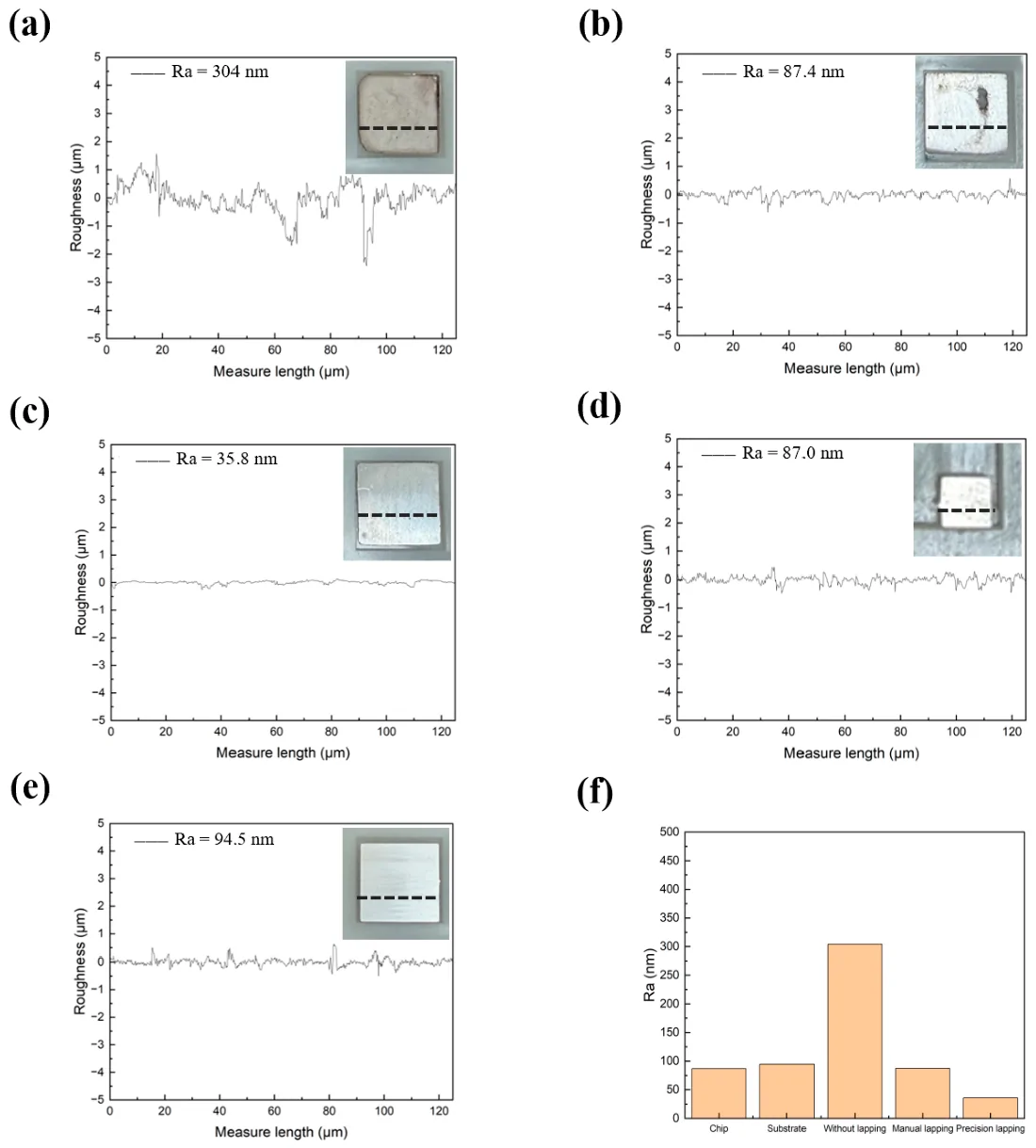
Surface roughness of the preforms with different surface states ((**a**) without lapping, (**b**) after manual lapping, and (**c**) after precision lapping), and surface roughness of (**d**) the Ag-electroplated Cu chip and (**e**) the Ag-electroplated Cu substrate. (**f**) Comparison of the measured R_a_ values.

Figure 5a–c show the surface roughness results measured for the Ag preforms prepared using different methods: hot pressing and cleaning (without lapping), manual lapping with 2000-grit sandpaper, and precision lapping. The without-lapping condition exhibited the highest Ra value of 304 nm, indicating a relatively rough surface state. This is interpreted as the result of the particle size of the BN particles used as a release agent, in addition to the Ag nanoparticles, which affected the surface roughness of the hot-pressed preform. In contrast, the Ra value decreased to 87.4 nm after manual lapping, and further decreased markedly to 35.8 nm after precision lapping, clearly demonstrating that a smooth surface free of coarse asperities was produced. Meanwhile, Figure 5d,e show the surface roughness of the electroplated Ag layers on the chip and substrate used for bonding; the Ra values were measured to be 87.0 nm and 94.5 nm, respectively, indicating similar surface states produced by identical electroplating. The measured Ra values are compared in Figure 5f. Because the Ra values of the chip and substrate were similar to each other, the surface of the manually lapped preform exhibited a roughness comparable to that of the chip and substrate, whereas the surface of the precision-lapped preform was far smoother and flatter. Considering that the main bonding mechanism during the subsequent pressure-assisted bonding is solid-state surface diffusion, such differences in the surface roughness of the Ag preform were expected to cause differences in the degree of sinter-bonding based on the actual interfacial contact area and conformity. Therefore, the shear strengths of the bond lines formed under identical pressures and temperatures were measured as a function of bonding time.

### 3.3. Sinter-Bonding Characteristics of Ag Preform

**Figure 6:**
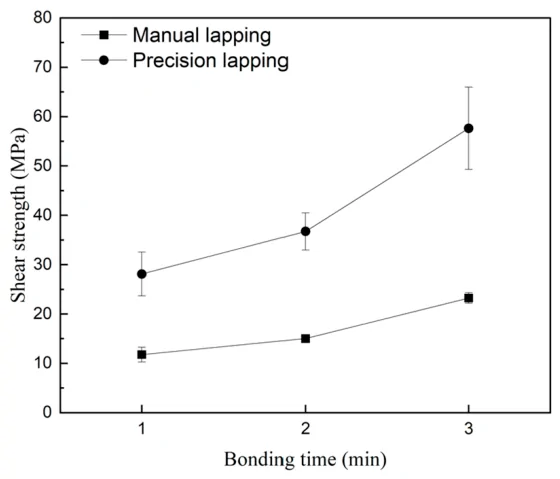
Shear strengths of the bond lines formed by sinter-bonding the Ag layer/Ag preform/Ag layer samples at 250 °C under 10 MPa in air, as a function of the surface lapping method of the preform and the bonding time.

Figure 6 shows the variation in the shear strength of the bond lines formed by interposing the fabricated Ag preform between the Ag-electroplated Cu chip and the Ag-electroplated Cu substrate and sinter-bonding at 250 °C under 10 MPa in air, as a function of the surface lapping method of the preform and the bonding time. For both the manually lapped and precision-lapped samples, the shear strength gradually increased with increasing bonding time (the unlapped samples are not plotted owing to their low strength values). However, the samples using the manually lapped preform exhibited generally low shear strengths of 11.77 (±1.50) MPa after 1 min and 15.00 (±0.72) MPa after 2 min, with a shear strength of 23.22 (±1.06) MPa measured after 3 min.

**Figure 7:**
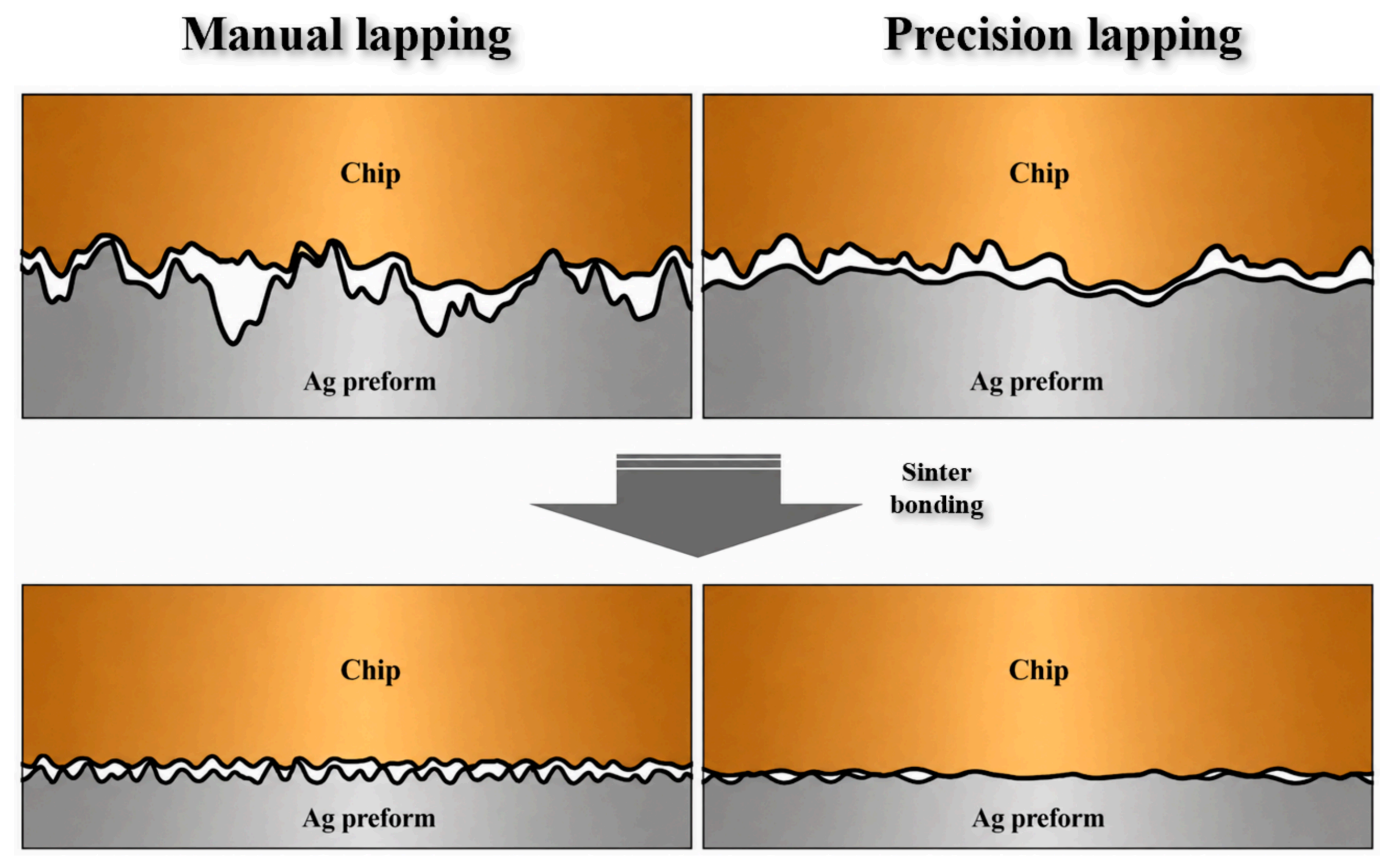
Schematic illustration of the progress of sinter-bonding at the chip/preform interface depending on the surface roughness of the preform.

As schematically illustrated in Figure 7, this was attributed to the insufficient mutual contact area and conformity at the interfaces, resulting from the similarity between the surface roughness of the preform and that of the interfacial Ag layers on the chip and substrate, which prevented effective sinter-bonding from being accomplished at the interfaces. That is, when the relatively high asperities of the interfacial Ag layers on the chip and substrate met the relatively high asperities on the preform surface, large surrounding regions may remain out of interfacial contact during the contact of these asperities. These regions may ultimately undergo ineffective sinter-bonding, resulting in low strength values.

In contrast, when the precision-lapped preform was used, a sufficient strength of 28.09 (±4.44) MPa was measured even after bonding for only 1 min, increasing to 36.72 (±3.75) MPa after 2 min and reaching a shear strength of 57.62 (±8.33) MPa after 3 min. This is interpreted as follows: Owing to the low surface roughness of the preform, the asperities of the relatively rough electroplated Ag layers on the chip and substrate touched the preform surface and were flattened by plastic deformation under external pressure, increasing the mutual contact area and conformity, which extended across the entire bonding interface.

As the bonding time increased, the surface and grain boundary diffusion of Ag through the interfacial contact areas and between the Ag particles within the preform proceeded continuously, leading to progressive microstructural coarsening of the Ag particles and a continuous reduction in the void area within the bond line [32,33]. Consequently, for sinter-bonding using a Ag preform, differences in the surface roughness of the preform induce substantial differences in interfacial sinter-bonding and ultimately act as a key factor governing the final bonding characteristics. In other words, excellent sinter-bonding performance can be realized by controlling the surface roughness of the Ag preform to a low level and then employing a sufficient bonding time. Meanwhile, the capping agent remaining from the preform fabrication stage was gradually removed by thermal decomposition during the heating stage of the subsequent sinter-bonding, enabling direct contact and diffusion between the Ag particles, thereby contributing to the formation of a dense Ag bond line.

### 3.4. Microstructural Characteristics of the Formed Bond Line

**Figure 8:**
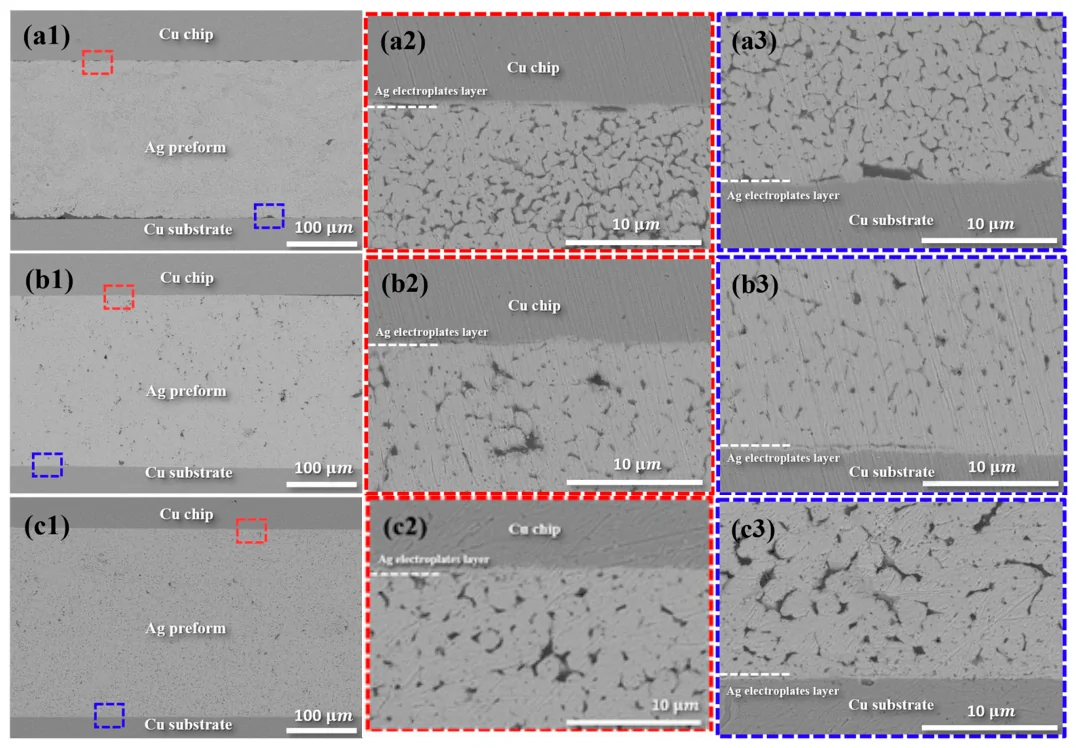
Cross-sectional BSE images of the bond lines formed using the Ag preforms after sinter-bonding for 1 min under 10 MPa at 250 °C in air with different preform surface preparations: (**a**) without lapping, (**b**) manual lapping, and (**c**) precision lapping. (**a1**−**c1**) Entire bond lines, (**a2**−**c2**) enlarged views of the upper Ag-electroplated Cu chip/Ag bond line interfaces, and (**a3**−**c3**) enlarged views of the lower Ag bond line/Ag-electroplated Cu substrate interfaces.

Figure 8 compares the cross-sectional BSE images of the bond lines formed for 1 min at 250 °C under 10 MPa in air using Ag preforms with different surface states. Figure 8(a1–c1) show the entire bond-line cross-sections, Figure 8(a2–c2) show enlarged views of the upper Ag-electroplated Cu chip/Ag bond line interfaces, and Figure 8(a3–c3) show enlarged views of the lower Ag bond line/Ag-electroplated Cu substrate interfaces. The images correspond to (a) without-lapping, (b) manual-lapping, and (c) precision-lapping conditions, respectively. Without lapping, the sintering did not proceed well at the upper and lower interfaces in Figure 8(a2,a3), and coarse voids elongated parallel to the interfaces were observed. Because the surface roughness of the preform was very large, when the high asperities on the preform surface touched the interfacial Ag layers on the chip and substrate, large-area regions surrounding the asperities remained out of interfacial contact. Owing to the large size of these asperities, interfacial contact did not develop around them even during the subsequent pressure-assisted sinter-bonding, leaving elongated coarse voids. In contrast, for the manually lapped preform (Figure 8(b2,b3)), the interfacial voids decreased, and the continuity of the interfacial bonding improved compared with the unlapped case; however, crack-like fine voids interconnected along the interfacial direction were observed, indicating that the non-contact state around the asperity-asperity contacts between the interfacial Ag layers and the preform surface was still influential. This indicates that, although lapping reduced the surface roughness of the preform, upper and lower asperities of a similar scale could still meet each other. Consequently, sinter-bonding through contact in the surrounding lower regions would remain very sluggish, potentially leading to reduced bonding strength. Finally, for the precision-lapped preform, the highest interfacial connection ratio and lowest bond-line porosity were observed, as confirmed in Figure 8(c2,c3), exhibiting high bonding uniformity where the boundary between the electroplated Ag layer and the Ag bond line was barely distinguishable. This is because, as the surface roughness of the preform decreased, the tips of the fine asperities on the relatively rough electroplated Ag layer were crushed and eliminated by plastic deformation under external pressure and heating, while the blunted asperities filled the surrounding void regions, reducing and isolating the residual voids. Diffusion-based sintering can then proceed effectively through the maximized contact area, thereby rapidly strengthening the interfacial regions.

Accordingly, as observed in Figure 8(c2,c3), the upper and lower interfacial regions exhibited higher densities than those of the interior of the bond line.

**Figure 9:**
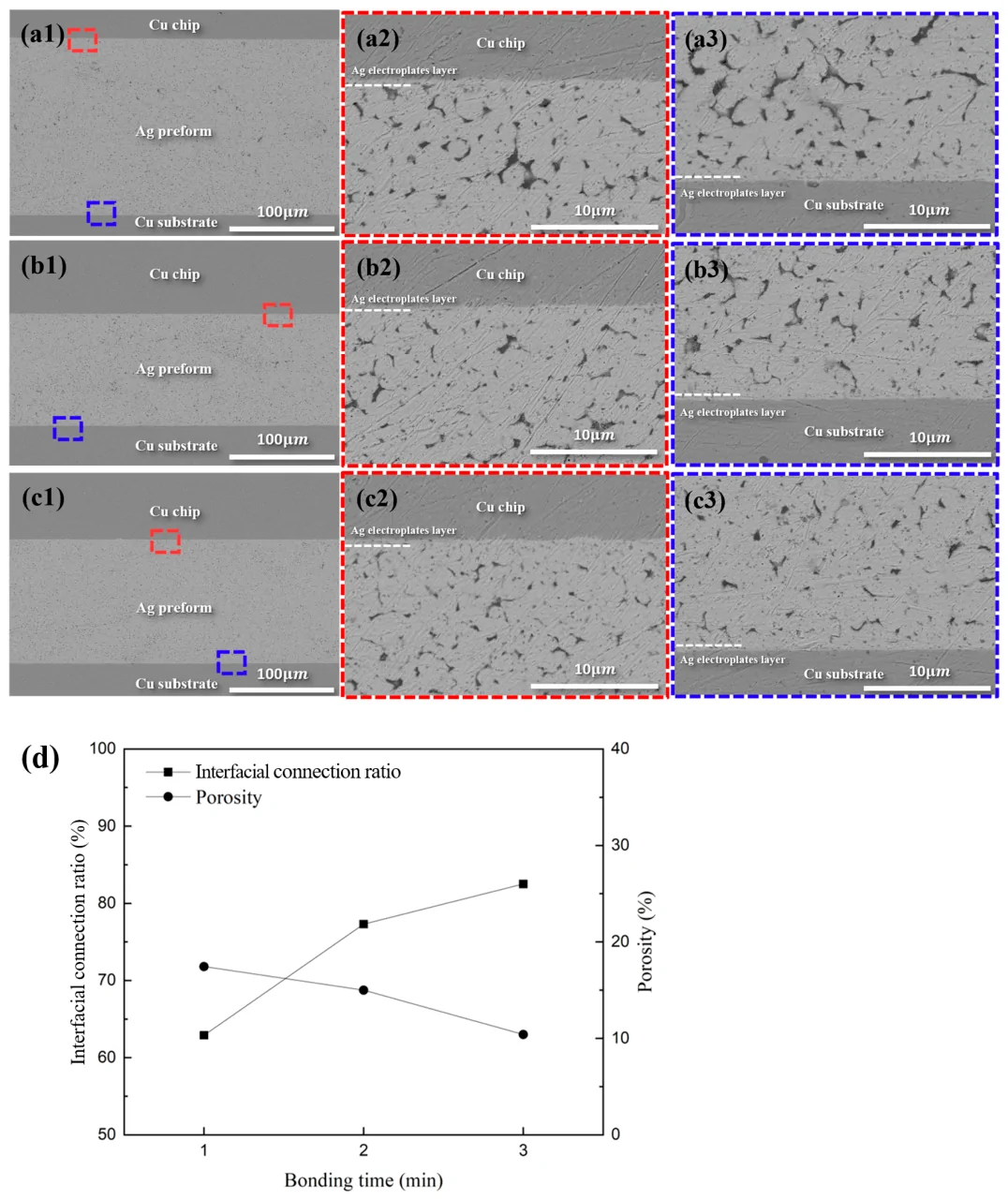
Cross-sectional BSE images of the bond lines formed with the precision-lapped Ag preform after sinter-bonding under 10 MPa at 250 °C in air for different bonding times: (**a**) 1 min, (**b**) 2 min, and (**c**) 3 min. (**a1**−**c1**) Entire bond lines, (**a2**−**c2**) enlarged views of the upper Ag-electroplated Cu chip/Ag bond line interfaces, and (**a3**−**c3**) enlarged views of the lower Ag bond line/Ag-electroplated Cu substrate interfaces. (**d**) Interfacial connection ratio and porosity of the bond lines with different bonding times.

Figure 9 compares the cross-sectional BSE images of the bond lines formed using the precision-lapped preform at 250 °C under 10 MPa in air as a function of bonding time. Figure 9(a1–c1) show the bond-line cross sections corresponding to bonding times of 1, 2, and 3 min. For the 1 min condition Figure 9(a1–a3), relatively numerous voids existed within the bond line, and fine voids and incompletely bonded regions were also observed at the upper and lower interfaces, indicating a somewhat insufficient degree of sintering. In contrast, when the bonding time was increased to 2 min Figure 9(b1–b3), the void area within the bond line decreased, and the density improved. This reduction in void area and increase in density are attributed to neck growth between the Ag particles during pressure-assisted sintering, together with the formation of new contacts and necks through local plastic deformation. The bonding states at the upper and lower interfaces also improved, with fewer fine voids and incompletely bonded regions. Finally, for the 3 min joints Figure 9(c1–c3), the void area decreased further, yielding the densest bond-line microstructure, and the continuous fine voids were eliminated at both the upper and lower interfaces, resulting in the most continuous bonding state. The improved continuity substantially enhanced the microstructural integrity of the weakest interfacial regions under shear testing. Figure 9d plots the interfacial connection ratio and bond-line porosity as functions of the bonding time. The interfacial connection ratio represents the degree of connection at the interface between the electroplated Ag layer and the Ag preform and was calculated from the SEM images using the following equation [34,35,36]:

δ = Σl_conttected interface_/l_interface_

Here, δ is the interfacial connection ratio, l_interface_ is the length of the interface, and l_connected interface_ is the total length of the connected regions within the interface. As expected, the connection ratio clearly increased, and the porosity decreased with increasing bonding time. The connection ratio remained at 62.9% after 1 min of bonding, increased to 77.3% after 2 min, and increased to 82.5% after 3 min. These values are higher than those obtained using Ag sinter pastes on Ag metallization and Au substrates and are comparable to the sinter-bonding performance of a Ag flake-based preform with ultrafine-grained surfaces, which exhibits an interfacial connection ratio of 84.1% after bonding for 3 min [35,37]. In addition, the porosity was as high as 17.44% after bonding for 1 min, but decreased to 15.00% after 2 min and further to 10.40% after 3 min. These changes reflect typical sintering behavior, in which interparticle diffusion, neck growth, grain coarsening, and pore elimination proceed continuously with increasing bonding time under pressure and heating. They explain the increase in shear strength with bonding time, as shown in Figure 6. In conclusion, in the pressure-assisted sinter-bonding process using the Ag preform, increasing the bonding time from 1 to 3 min induced densification of the bond line and eliminated continuous fine voids at the interfacial regions, contributing to the formation of high-quality joints with low bond-line porosity and strong interfacial bonding states.

**Figure 10:**
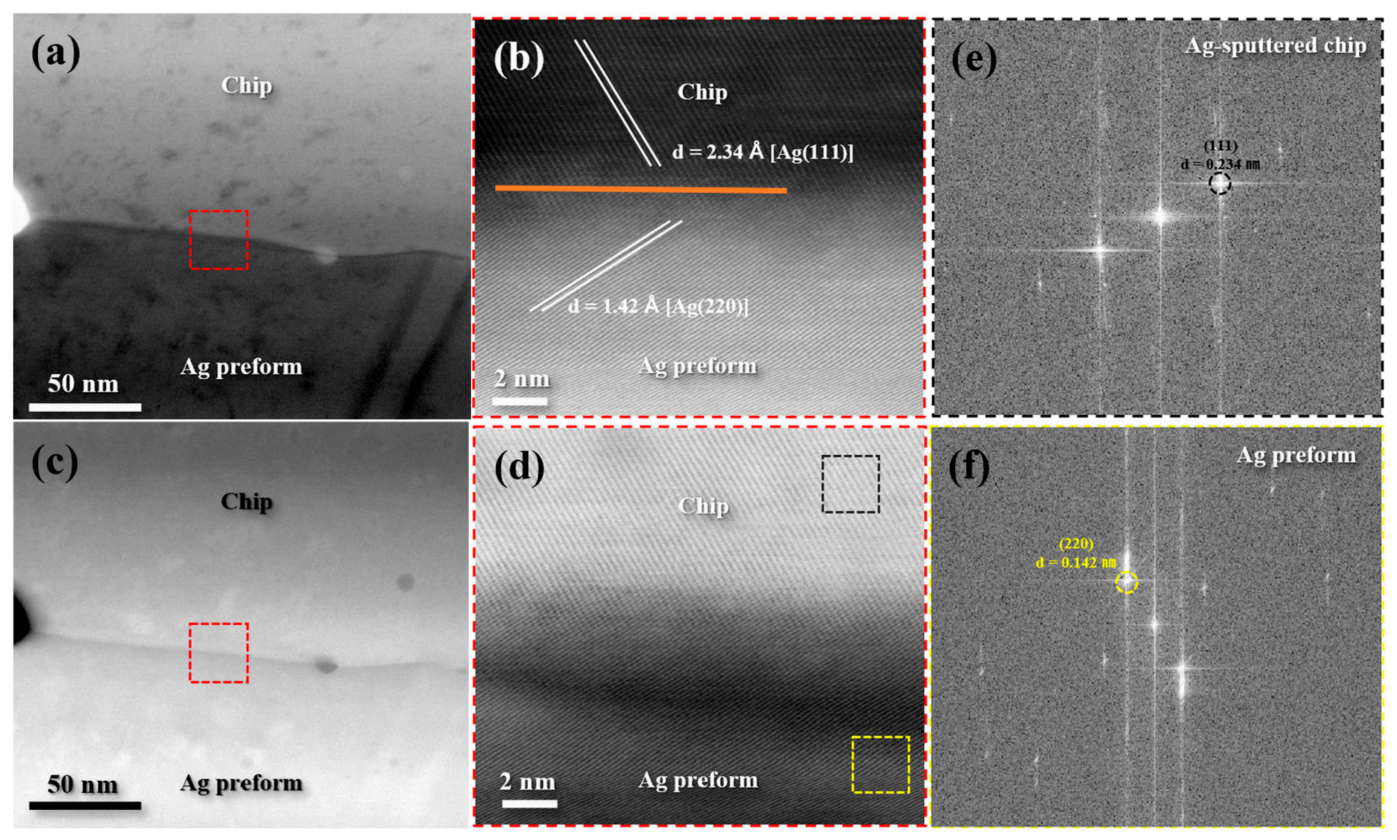
(**a**,**b**) High-resolution TEM images, (**c**,**d**) HAADF-STEM images (the rectangular dashed region in (**a**) corresponds to (**b**), while the rectangular dashed region in (**c**) corresponds to (**d**)), and (**e**,**f**) FFT patterns of the Ag-electroplated Cu chip/Ag bond line interface (the upper region corresponds to (**e**), while the lower region corresponds to (**f**,**d**) after sinter-bonding for 1 min under 10 MPa at 250 °C in air using the precision-lapped Ag preform.

Figure 10a–d show the high-resolution TEM and HAADF-STEM analysis results of the Ag-electroplated Cu chip/Ag bond line interfacial region of the sample sinter-bonded for 1 min at 250 °C under 10 MPa in air using the precision-lapped preform. Figure 10b,d show high-magnification images of the dotted rectangular regions indicated in Figure 10a,c, respectively. In the bonded region between the electroplated Ag layer beneath the chip and the bond line formed from the Ag preform Figure 10b, no distinct boundary layer was observed. Instead, an interfacial structure in which the Ag atomic arrangements were continuously connected was observed. That is, the lattice of the upper Ag layer, exhibiting the (111) plane, and the lattice of the lower bond line, exhibiting the (220) plane, faded out and gradually transformed as they approached the boundary and remained interconnected, indicating that the boundary was in a state of metallurgical bonding formed by the interdiffusion of Ag atoms. Furthermore, given that HAADF imaging produces an atomic-number-sensitive contrast between regions with different atomic distributions, the absence of any abrupt contrast change in the boundary region in Figure 10c indicates that the boundary was formed from the same Ag atoms; that is, the interfacial bonding proceeded through the mutual diffusion of Ag. Similarly, in the enlarged image in Figure 10d, only a continuous distribution of Ag atoms was observed at the boundary without the generation of voids or oxide layers, demonstrating that atomic-scale diffusion bonding between the electroplated Ag layer and Ag preform was effectively accomplished during sinter-bonding. Meanwhile, Figure 10e,f show the FFT patterns obtained from the upper dotted rectangular region corresponding to the Ag-electroplated layer beneath the chip and the lower dotted rectangular region corresponding to the Ag bond line in Figure 10d, which were indexed to the (111) and (220) planes of FCC Ag, respectively. The FFT pattern observed in the bond-line region exemplifies the crystallographic planes observed in the bulk-like high-density structure formed as the Ag preform progressively densified during pressure-assisted sintering. These atomic-scale microstructural analyses demonstrate that the fabricated Ag preform realized Ag/Ag bonding with the Ag layers of the chip and substrate through effective atomic diffusion during pressure-assisted sinter-bonding, forming a stable metallic bond in which the distribution of Ag atoms was continuous across the boundary despite the different lattice planes and orientations of the upper and lower Ag. Moreover, the absence of oxide formation at the interface, even though the bonding was performed in air, is attributed to the thermodynamic nature of Ag, which does not form oxides at the bonding temperature of 250 °C [38,39]. Consequently, the present Ag preform demonstrates potential as a die-attach material for high-temperature applications, capable of forming excellent interfacial microstructures and highly reliable joints during pressure-assisted sinter-bonding with Ag-electroplated chips and substrates.

### 3.5. Fractography

**Figure 11:**
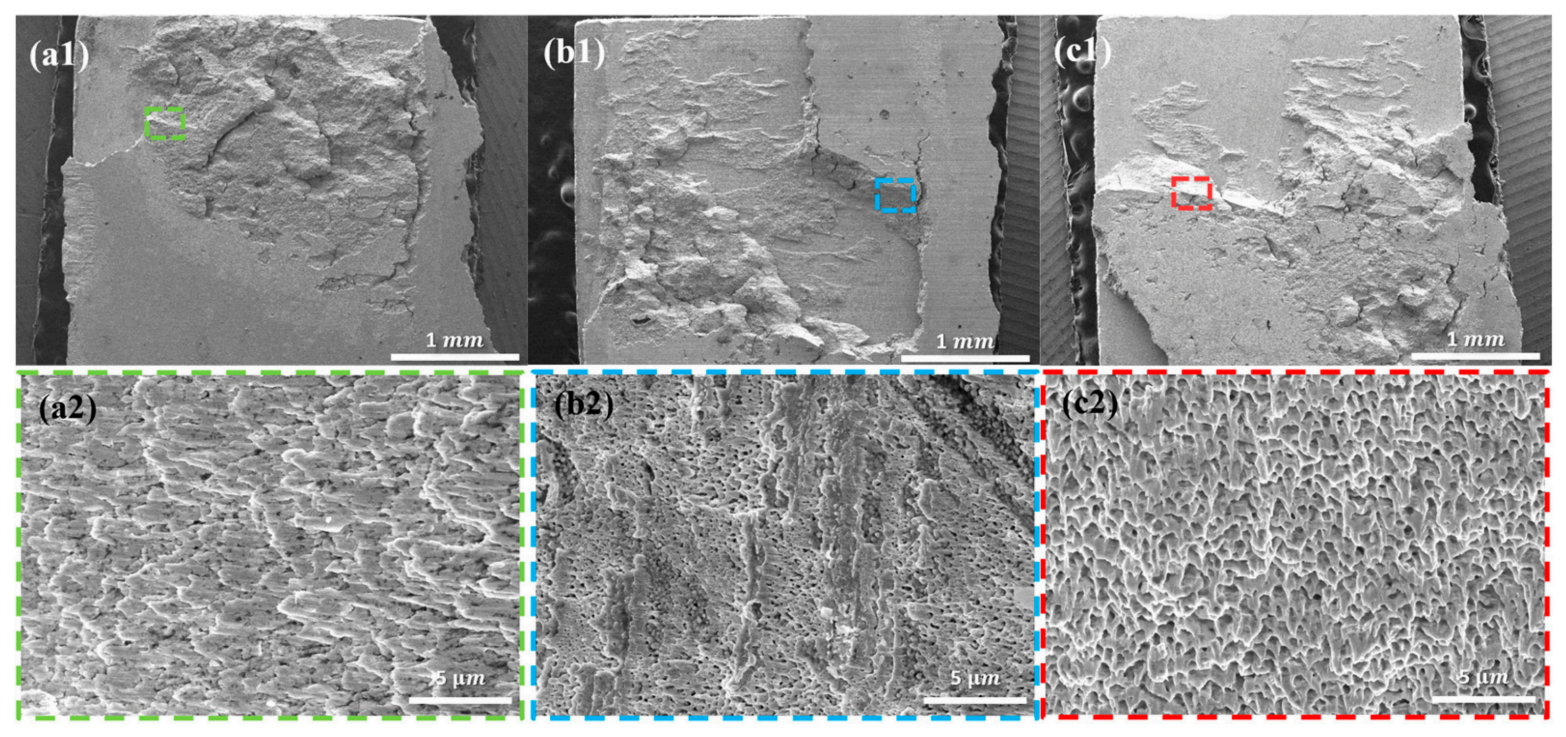
SEM images of the fracture surfaces on the chip side after shear testing of the chip/precision-lapped Ag preform/substrate sandwich specimens sinter-bonded under 10 MPa at 250 °C in air for different bonding times: (**a**) 1 min, (**b**) 2 min, and (**c**) 3 min. (**a1**−**c1**) Low-magnification images and (**a2**−**c2**) enlarged views of the dotted rectangular regions in (**a1**−**c1**).

Figure 11 shows the SEM fracture surfaces on the chip side, arranged by the bonding time, observed after shear testing of the chip/precision-lapped Ag preform/substrate samples sinter-bonded at 250 °C under 10 MPa in air. The upper images Figure 11(a1–c1) show low-magnification views of the entire fracture surface, whereas the lower images Figure 11(a2–c2) show enlarged views of the dotted rectangular regions in the upper images. In all samples bonded for 1–3 min, fracture occurred along the interior of the bond line or propagated through the bond-line interior, together with the upper and lower interfaces, confirming that cohesive failure was the dominant failure mode. This indicates that sufficiently strong interfacial bonding was formed between the upper and lower Ag plating layers and the Ag preform. The enlarged fractographs in Figure 11(a2–c2) show that even after only 1 min of bonding, parts of the fracture surface exhibited highly stretched metallic ligaments rather than retaining the original particle morphology, indicating extensive plastic deformation prior to failure. This suggests that a sound-bonded microstructure with sufficient neck formation between the Ag particles in the preform was formed despite the short bonding time. Nevertheless, although the average shear strength was 28.09 MPa, the interfacial bonding remained partially insufficient, resulting in a large interfacial fracture area. As the bonding time increased to 2 and 3 min, the porous structures observed on the fracture surfaces became finer and more refined as a result of the denser microstructure, and the greater number of stretched metallic ligaments per unit area implied a fracture behavior characterized by more pronounced and stable plastic deformation. The interfacial fracture area also tended to decrease further in the 3 min sample compared with that in the 2 min sample. These changes in the fracture-surface microstructure represent the increased sintering between the Ag particles within the preform and at the Ag layer/Ag preform interfaces, and correspond well to the aforementioned high shear strength results.

## 4. Conclusions

A binder-free porous Ag preform was fabricated by direct hot-pressing of quasi-spherical Ag nanoparticles (70.2 nm) synthesized by a wet-chemical method at 200 **°**C for 1 min under 10 MPa. The sinter-bonding characteristics of the Ag-electroplated Cu chips and substrates were evaluated at 250 **°**C under a pressure of 10 MPa in air. The hot-pressed preform exhibited a uniform porous network structure, in which the nanoparticles were sintered only to the extent of neck formation, thereby retaining high sintering activity while enabling local plastic deformation for conformal contact with the counterpart faying surfaces. The surface roughness of the preform was found to be a key factor governing the bonding performance. When the preform surface was rendered smoother (Ra = 35.8 nm) than the electroplated Ag layers of the chip and substrate by precision lapping, the counterpart asperities were plastically flattened under the applied pressure, thereby maximizing the real interfacial contact area. Consequently, shear strengths of 28.1, 36.7, and 57.6 MPa were achieved after bonding for only 1, 2, and 3 min, respectively, with the interfacial connection ratio increasing from 62.9% to 82.5% and the bond-line porosity decreasing to 10.4%. TEM and HAADF-STEM analyses verified that oxide- and void-free metallurgical bonding was accomplished through the interdiffusion of Ag atoms across the preform/Ag layer interface within only 1 min, and fractographic analyses confirmed cohesive failure accompanied by highly stretched metallic ligaments. These results demonstrate that the direct consolidation of Ag nanoparticles provides a simple, binder-free route to preform-type die-attach materials, enabling rapid, low-pressure sinter-bonding and offering strong potential for highly reliable packaging applications involving SiC power devices.

## Data Availability

The original contributions presented in the study are included in the article, and further inquiries can be directed to the corresponding author.
